# Determining the efficacy of camera traps, live capture traps, and detection dogs for locating cryptic small mammal species

**DOI:** 10.1002/ece3.5972

**Published:** 2020-01-08

**Authors:** Morgan L. Thomas, Lynn Baker, James R. Beattie, Andrew M. Baker

**Affiliations:** ^1^ School of Earth, Environmental and Biological Sciences Science and Engineering Faculty Queensland University of Technology Brisbane Qld Australia; ^2^ Canines for Wildlife Brierfield NSW Australia; ^3^ Research School of Astronomy and Astrophysics Australian National University Canberra ACT Australia; ^4^ Biodiversity Program Queensland Museum South Brisbane Qld Australia

**Keywords:** *Antechinus arktos*, black‐tailed dusky antechinus, camera trapping, effectiveness, live trapping

## Abstract

Metal box (e.g., Elliott, Sherman) traps and remote cameras are two of the most commonly employed methods presently used to survey terrestrial mammals. However, their relative efficacy at accurately detecting cryptic small mammals has not been adequately assessed. The present study therefore compared the effectiveness of metal box (Elliott) traps and vertically oriented, close range, white flash camera traps in detecting small mammals occurring in the Scenic Rim of eastern Australia. We also conducted a preliminary survey to determine effectiveness of a conservation detection dog (CDD) for identifying presence of a threatened carnivorous marsupial, *Antechinus arktos*, in present‐day and historical locations, using camera traps to corroborate detections. 200 Elliott traps and 20 white flash camera traps were set for four deployments per method, across a site where the target small mammals, including *A. arktos*, are known to occur. Camera traps produced higher detection probabilities than Elliott traps for all four species. Thus, vertically mounted white flash cameras were preferable for detecting the presence of cryptic small mammals in our survey. The CDD, which had been trained to detect *A. arktos* scat, indicated in total 31 times when deployed in the field survey area, with subsequent camera trap deployments specifically corroborating *A. arktos* presence at 100% (3) indication locations. Importantly, the dog indicated twice within Border Ranges National Park, where historical (1980s–1990s) specimen‐based records indicate the species was present, but extensive Elliott and camera trapping over the last 5–10 years have resulted in zero *A. arktos* captures. Camera traps subsequently corroborated *A. arktos* presence at these sites. This demonstrates that detection dogs can be a highly effective means of locating threatened, cryptic species, especially when traditional methods are unable to detect low‐density mammal populations.

## Graphical abstract

Studies comparing detection methods are imperative to the development and improvement of conservation and management strategies. Camera traps provide higher detection probabilities, when compared to Elliott traps, and are time invariant. Similarly, the detection dog demonstrated high levels of success, locating the target species, *Antechinus arktos*, in a location that it has not been recorded in 30 years.

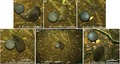

## 1 INTRODUCTION

Current global biodiversity losses and coinciding increased rates of extinction emphasize the need to develop our knowledge surrounding species occurrence so that strategic and effective global conservation programs can be developed (Barnosky et al., 2011; Bilney, 2014; Woinarski, Burbidge, & Harrison, 2015; McCallum, 2015). Successfully determining the occurrence/detection of a given species ultimately contributes to the development of tailored conservation, management, and monitoring programs, which can help alleviate extinction risk. Specifically, by effectively surveying species residence within a landscape, it permits better understanding of the species’ dynamics, movements, and status. How these factors are affected by other ecological variables can also be examined, and consequently, this enables development of strategic conservation and management programs, which augment efforts to conserve target species (Diggins, Gilley, Kelly, & Ford, 2016). Improving our capability to detect rare and elusive species is clearly fundamental to the success of such programs (Diggins et al., 2016). However, dependable information on the location of a species can be difficult to obtain, especially for cryptic and hard to detect taxa (Paull, Claridge, & Cunningham, 2012; Vine et al., 2009).

A variety of different sampling methods exists for surveying terrestrial mammals; however, the type of detection method(s) employed can have significant implications for data quality and survey success (Thompson & Thompson, 2007). Direct methods requiring the physical, live capture of an individual are often used for detection of small‐ and medium‐sized terrestrial mammals and have been effectively employed for estimating species richness and abundance (Read, 1988; Tasker & Dickman, 2001). Live capture allows for the collection of unambiguous data and facilitates collection of further ecological and morphological information, including sex, age, and reproductive condition (De Bondi, White, Stevens, & Cooke, 2010; González‐Esteban, Villate, & Irizar, 2004). However, while broadly successful, live trapping may not be as useful for rare and elusive species, which have lower capture probabilities (Jones, McShea, Conroy, & Kunz, 1996). Such direct detection methods can also often be labor, time, and cost intensive, which has implications for the quantity and delivery of resulting data (Stanley & Royle, 2005; Wiewel, Clark, & Sovada, 2007). Live capture methods also have a drawback for estimating detectability when compared to automated recording units (i.e., wildlife cameras) as live traps are limited to detecting one individual per sampling occasion, whereas noninvasive detection methods such as wildlife cameras can detect multiple individuals per sampling occasion.

Camera trapping, a noninvasive, indirect detection method, has shown substantial promise for detecting small‐ and medium‐sized mammals since its inception (Griffiths, 1994; Karanth, 1995; McCallum, 2013; Rovero, Zimmermann, Berzi, & Meek, 2013). Camera trapping involves deploying remotely triggered camera units, which capture photographs and/or videos of a passing animal (Rovero et al., 2013; Rowcliffe, Field, Turvey, & Carbone, 2008). Camera traps, unlike live trapping methods, can operate 24 hr a day and may run for extended periods in a variety of landscapes and weather conditions (González‐Esteban et al., 2004; Peterson & Thomas, 1998; Vine et al., 2009). Camera trapping ultimately removes the need to physically handle an individual and offers a means of detecting rare, elusive, or trap‐shy individuals that may be missed by traditional, intensive, shorter‐duration live trapping methods (Gray, Dennis, & Baker, 2017; Rendall, Sutherland, Cooke, & White, 2014).

Typically, camera traps are mounted horizontally, with the lens oriented outwards, which has been successful for surveying various large‐ and medium‐sized mammals (Maffei, Noss, Cuéllar, & Rumiz, 2005; Rovero et al., 2017; Trolle, Noss, Cordeiro, & Oliveira, 2008). Recently, several studies have demonstrated the success of a modified camera trap mounting design for use in small mammal surveys. De Bondi et al. (2010) and Gray, Dennis, et al. (2017) demonstrated that infrared camera traps mounted vertically, with the lens oriented toward the ground at close range (<1.5 m), could be successfully used to detect and identify morphologically similar small mammal species. However, accurately identifying individuals to species, especially with infrared cameras, can still be problematic and time consuming, especially for species displaying morphological similarities and/or those with predominantly nocturnal behaviors (De Bondi et al., 2010; Meek & Pittet, 2012; Meek, Vernes, & Falzon, 2013).

An infrared camera flash is less detectable by species when images are taken compared with white flash (Meek & Pittet, 2012). The discreetness of infrared cameras is a result of scarcely observable light that is produced by a selection of light emitting diodes (LED) when an image is taken, triggered by changes in infrared light in the detection zone (Trolliet, Huynen, Vermeulen, & Hambuckers, 2014). However, infrared cameras only produce black and white, and potentially blurred photos, which may result in the inability to correctly identify the photographed species (Glen, Cockburn, Nichols, Ekanayake, & Warburton, 2013; Trolliet et al., 2014).

In such cases, the use of white flash cameras instead of infrared cameras may improve identification accuracy, since the former provide colored photographs (even at night) of a much higher quality (Meek & Pittet, 2012). White flash cameras also offer the prospect of improving speed (and therefore cost‐effectiveness) of post‐image analysis because of the clarity and color, which allows for faster identification of an individual, especially for species sharing similar traits. Processing time can be a significant drawback for indirect methods that require the user to search through thousands of images or videos (Glen et al., 2013).

In the last decade, conservation detection dogs have similarly provided an accurate, cost‐ and time‐effective method of detection for a range of target species (Akenson, Henjum, Wertz, & Craddock, 2001; Arnett, 2006; Browne, Stafford, & Fordham, 2015; Cristescu et al., 2015; Reindl‐Thompson, Shivik, Whitelaw, Hurt, & Higgins, 2006). This indirect detection technique is especially promising for locating threatened or rare species, which may display low densities or clustered populations (Hurt & Smith, 2009; Leigh & Dominick, 2015). Detection dogs have the potential to reduce reliance on other traditional methods such as live trapping. Detection dogs also provide an opportunity for a preliminary survey to determine presence or absence of a species and may be employed to locate the most appropriate specific locations for subsequent trapping and survey efforts (Reed, Bidlack, Hurt, & Getz, 2011). Detection dogs have been successfully used to detect a range of medium–large target mammals (e.g., bobcats, bears, koalas, etc.; Harrison, 2006; Long, Donovan, Mackay, Zielinski, & Buzas, 2007; Romane et al., 2015). However, their utility for detecting smaller, cryptic, and rare mammal species, such as threatened rodents or marsupials, is currently being explored in a range of settings to serve as models for the approach going forward.

The focus of the present study is the black‐tailed Dusky Antechinus, *Antechinus arktos*, a small, recently discovered, federally endangered carnivorous marsupial mammal, located in subtropical rainforests of mid‐eastern Australia (Baker, Mutton, Hines, & Dyck, 2014; Van Dyck & Strahan, 2008). The species’ distribution is severely restricted, apparently limited to the highest altitude peaks (950+ m altitude) within the Tweed Shield Volcano caldera.

In a suite of foundational ecological studies, Gray, Baker, and Firn (2017), Gray, Burwell, and Baker (2016), and Gray, Dennis, et al. (2017) assessed the breeding synchrony, growth, and detectability of *A. arktos*. The latter study assessed the feasibility of close range, vertically oriented, baited infrared cameras to detect *A. arktos*. Gray, Dennis, et al. (2017) concluded that the camera orientation successfully provided close‐up images that allowed for species‐level discrimination. Both *A. arktos* and the co‐occurring Brown Antechinus, *Antechinus stuartii*, can typically be distinguished by their differences in size (*A. arktos* are larger), pelage color (*A. arktos* are darker), and tail (*A. arktos* have a thicker, blacker tail). However, *A. arktos* is mostly nocturnal and returned images in the study of Gray, Dennis, et al. (2017) were black and white. Thus, a combination of stills and video was required to permit species‐level discrimination between the rare *A. arktos* and its (orders of magnitude) more abundant congener, *Antechinus stuartii*. This process proved arduous and inefficient when multiplied across thousands of records. Gray, Dennis, et al. (2017) did not specifically compare efficacy of live trapping versus cameras for detecting small mammals, including *A. arktos*. They nevertheless suggested that although both methods had merit, live trapping may be preferable given the challenges inherent in post‐deployment image handling and the need to rebait cameras every third day. Other recent studies have also suggested that a combination of detection methods may deliver the most effective and cost‐efficient method for locating a target species (Garden, McAlpine, Possingham, & Jones, 2007; Paull et al., 2012).

The ability to accurately and effectively locate a species is imperative to the successful development of any conservation or management strategy, especially for rare or cryptic species. The current study therefore sought to extend previous detection studies by employing vertically oriented, close range, white flash cameras and specifically compare the results obtained with those of live capture (Elliott) traps in detecting the presence of a suite of small mammals in the Tweed Volcano caldera, including the endangered *A. arktos*. Further, we wanted to investigate the utility of a detection dog in detecting the presence of *A. arktos*.

Specifically, we aimed to:

1. Compare the effectiveness of Elliott traps and vertically mounted white flash camera traps at a site level, using overall detection probabilities to establish the most effective means of detecting the presence of small mammals, including *A. arktos*;
2. Conduct a preliminary survey of the effectiveness of a conservation detection dog for identifying the broad‐scale presence of *A. arktos* in several present‐day and historical locations, using camera traps to attempt to corroborate any canine‐based detections;
3. Use the present research model as a basis to discuss the utility of the various detection techniques for broader use in projects designed to detect the presence of cryptic/rare small mammal species.

**Figure 1:**
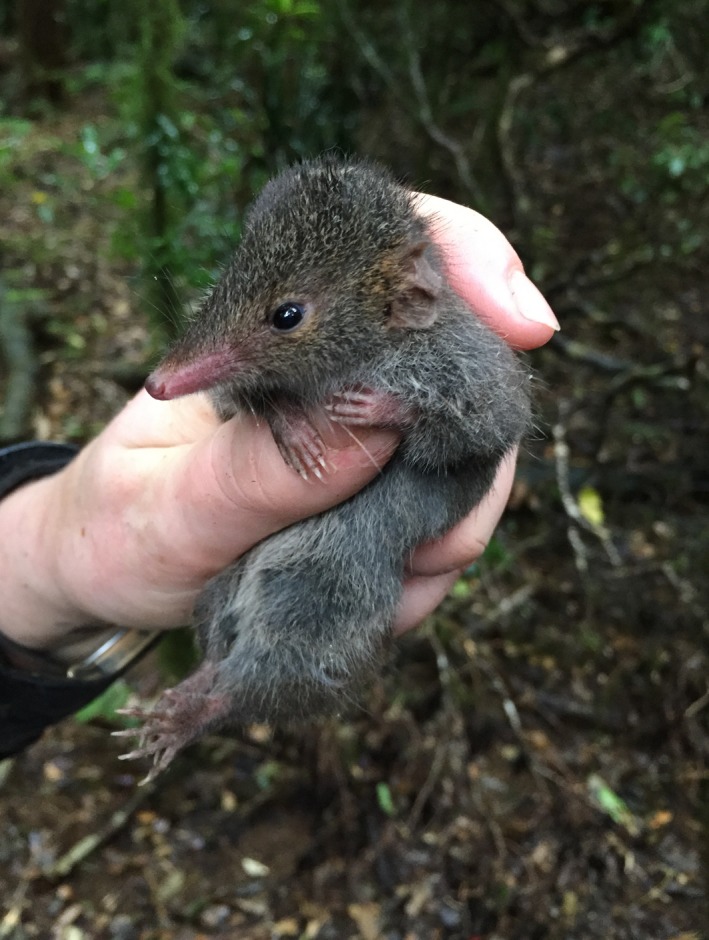
Antechinus arktos

We hypothesized that vertically mounted, white flash camera traps would provide a more effective means of detecting the presence of small mammal species at a given site, when compared with live trapping (Elliott) methods. Further, we hypothesized that the detection dog would effectively detect *A. arktos* in known locations (Figure 1).

## 2 MATERIALS AND METHODS

The study was conducted with approval from the Queensland University of Technology Ethics Committee (approval number: 1700000210), Queensland Parks and Wildlife Service (approval number: WITK18548417), and New South Wales Parks and Wildlife Service (approval number: Sl101881).

### 2.1 Study site

Live trapping and camera trapping programs were undertaken at Best of All Lookout (28.2415°S, 153.2640°E), within Springbrook National Park, located in the Gold Coast hinterland, ~110 km south of Brisbane, Queensland, mid‐eastern Australia. Springbrook National Park is one of four national parks located on the edge of the Tweed Shield Volcano caldera and harbors remnants of UNESCO World Heritage listed “Gondwana Rainforests of Australia” (Hunter, 2003). The present study used a single overall site containing a pre‐existing trapping grid from ongoing parallel research involving *A. arktos* because the species is known at that location.

Detection dog components of the present work were conducted at a broader scale throughout Springbrook National Park, around and within the live trapping/camera trapping regime areas, and within Border Ranges National Park. Border Ranges National Park is located in the scenic rim, ~140 km south of Brisbane, at the edge of the Tweed Shield Volcano caldera, to the southwest of Springbrook National Park. Border Ranges National Park also exhibits major remnants of UNESCO World Heritage listed “Gondwana Rainforests of Australia.” Border Ranges National Park represents a historical site for *A. arktos*, where confirmed voucher specimen‐based records were last retrieved in the late 1980s/early 1990s (Baker et al., 2014). In the present study, detection dog surveys made use of existing trapping grids at both national parks, along with historical capture sites of *A. arktos*, and sites displaying ostensibly suitable habitat and/or climate requirements (see Gray, Baker, et al., 2017; Gray et al., 2016; Gray, Dennis, et al., 2017).

### 2.2 Experimental design

Camera traps and Elliott traps were deployed across the same spatial scale (the pre‐existing trapping grid in Springbrook National Park), which allowed for comparison of detection methods within similar environmental conditions. This experimental design was selected because it attempted to explore the effectiveness of each method, while also maximizing captures and minimizing stress on captured animals (De Bondi et al., 2010; Tasker & Dickman, 2001). It also allowed for a direct comparison with previous live trapping and camera trapping efforts in the same location. This meant that although displaying different temporal scales, we could compare the effectiveness within the same area under the same conditions and attempt to account for the difference in sampling effort between the two methods.

### 2.3 Live trapping

Live trapping was conducted across four deployments between 24 June and 18 August 2017 to target the period prior to breeding (September) for *A. arktos* (Gray, Baker, et al., 2017). A total of 200 type A aluminum Elliott folding traps (Elliott Scientific Equipment, Upwey, Vic.) were employed for the live capture of individuals per deployment, as per previous work on the species (Gray, Baker, et al., 2017; Gray Burwell & Baker, 2016). Elliott traps were stationed over a single pre‐existing trapping grid at Springbrook National Park, which consisted of four parallel transects (200 m total ~20 m apart). Each of the four transects had 25 tags placed ~8 m apart along their length, and each of these 25 tag locations was considered an individual site during analysis (Gray, Baker, et al., 2017; Gray, Dennis, et al., 2017). Two traps were set at each of the 25 tags, no <1 m apart from each other, along each transect (50 traps per transect) to increase chances of detection in an area typically displaying high levels of small mammal diversity (Dickman, 1986). Best of All Lookout is a known habitat of *A. arktos*, and the adopted trapping grid has been employed in previous studies involving live capture and camera trapping of *A. arktos*, making it ideal for the present work (Gray, Baker, et al., 2017).

*Antechinus arktos* is predominantly nocturnal (Gray, Dennis, et al., 2017), and thus, each individual deployment consisted of three consecutive nights. A mixture of peanut butter, oats, vegetable oil, and bacon was used for bait, which was replenished daily. Traps were set at sundown and cleared as close to sunrise as possible to minimize containment time of individuals and reduce potential overheating/exposure. Traps were left closed during daylight hours, and bait was replenished each night as necessary. All individuals were identified to species level and released immediately at point of capture. All *A. arktos* were weighed to the nearest 0.5 g using a Pesola spring balance, sexed, ear clipped, assessed for reproductive condition, and microchipped with a passive integrated transponder (PIT) tag for recapture ID. *Antechinus stuartii* were also weighed, sexed, and assessed for reproductive condition.

### 2.4 Camera trapping

Camera trapping was conducted across four deployments between 30 June and 21 August 2017 to target the period prior to breeding for *A. arktos* (Gray, Baker, et al., 2017), as for the live trapping component of this study. Each site was trapped commencing one night after live trapping events to allow individuals a period of regular foraging. Modified Reconyx PC850 Hyperfire Professional White Flash Camera Traps were used. Camera traps were set for three consecutive nights per deployment, as in the live trapping component of the study. Camera trap focal length was factory pre‐set to 70 cm and the detection grid altered to match this shorter focal length. One camera was set at every fifth tag along each of the same four transects used in the live trapping component of the study, and thus, a total of 5 cameras were spread evenly for each of the four transects, constituting 20 cameras in total across the trapping grid. This represents a small spatial scale to deploy cameras; however, *A. arktos* displays a very patchy and limited distribution in steep, difficult‐to‐traverse terrain (Gray, Baker, et al., 2017). The utilization of the single trapping grid composed of four transects at Springbrook National Park for both components also facilitated comparison of detection methods, as they were deployed in the same area, within days of each other.

**Figure 2:**
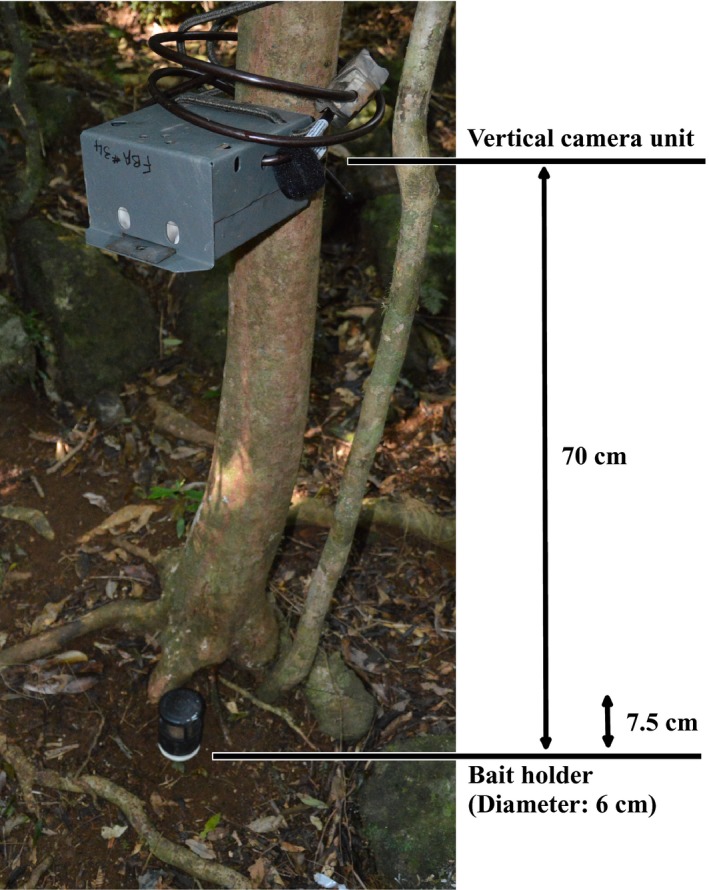
Schematic diagram of digital white flash camera setup, showing vertical positioning on a tree, with relevant distances and components labeled

In previous studies, cameras were typically mounted horizontally, often attached to structures such as trees, to effectively capture animal profiles (Sweitzer, Vuren, Gardner, Boyce, & Waithman, 2000). For the current study, however, we employed a revised camera mounting strategy to effectively detect small terrestrial mammal species, modifying the design of Gray, Dennis, et al. (2017). Camera units were mounted vertically on trees at a height of 70 cm, to produce photographs of the zone of ground directly underneath the camera (Figure 2) and attempt to correlate camera height with species body size/mass. To reduce the potential for false triggers and ensure unobstructed photographs, vegetation and dense leaf litter around the detection grid were removed prior to deployment.

Camera units were set (running) for the entire three‐day deployment, recording three photographs for every trigger, with a one‐second interval between each photograph in the same trigger event. These three photos were subjectively defined as a single photographic “event.” These settings were similarly selected to provide photographs of the individual in different positions and ultimately increase identification accuracy. Camera units were operational 24 hr per day, for each day of field deployment to account for potential diurnal activity (Gray, Baker, et al., 2017). When processing photographs, once a species had been recorded, individuals of the same species were not recorded for another 10 min to minimize the possibility of detecting the same individual again and maximize the potential to record individuals that rarely visited camera units (Gray, Dennis, et al., 2017; Weerakoon et al., 2014). Photographic events were recorded as binary response variables: “1” was used to indicate that the species had been observed and “0” used if the species was not observed. Data were collected in this manner for each camera, for each of the days across the entire three‐day deployment period (i.e., all photographic events taken were recorded this way except for individuals seen within 10 min of each other). Data were then pooled for each deployment at the trap level such that all data from day one to three of each deployment were pooled to indicate that the species was detected or not detected for that deployment. We acknowledge that this does not fully account for potential camera “recaptures” (i.e., individuals that may reappear at various times in the night under the same or different cameras). Similarly, our analysis could not account for recaptures of individuals of various species (other than PIT‐tagged *A. arktos* in Elliott traps), either between nights or deployments.

Bait used for the camera units was identical to that used in live trapping methods (see above). Bait containers were composed of one 50‐mm mosquito proof vent cowl (*W*: 62 mm, *H*: 84 mm) and one greywater hose adaptor (*W*: 57 mm, *H*: 62 mm) purchased from Bunnings Warehouse (Brisbane, Australia). These were spray‐painted black (to prevent under‐exposed photos) and secured to the ground using three, large tent pegs. This bait container set up allowed for individuals to view and smell the bait but not remove it; therefore, bait did not have to be replenished during each deployment. It also assisted with identification of individuals because of the known dimension of each bait container (62 mm diameter).

Photographs were processed, and all individuals were identified to species level based on pelage color/appearance, body size and shape, head size and shape, and tail length/appearance. If identification was not possible because of blurred images or lack of distinguishable features, it was classified as “unknown” and not used in subsequent analyses. A second examiner ensured identification accuracy.

### 2.5 Detection dog

The conservation detection dog was trained by a certified professional dog trainer (Certification Council for Professional Dog Trainers (CCPDT)). Dog obedience and socialization training began at nine weeks old, with odor detection training on *A. arktos* scat commencing at 12 months of age. The dog was trained to associate the scent of *A. arktos* with a reward (e.g., food, tennis ball) and to freeze (“indicate”) at the location of the odor. The dog was trained on a few *A. arktos* scats from previous live trapping studies at the current study site, and again trained for one month after fresh scat, hair and live trap bedding material used by captured *A. arktos* were obtained in July 2017. The detection dog passed a certification test for *A. arktos*, which included flush control tests, obedience tests, and odor detection tests, before being deployed for field training and validation. Further reaffirmation of the dog's training occurred through double‐blind tests in the field, resulting in 100% of indications being correct, with no false positives (validated by camera traps postsurvey).

Field trials were conducted across multiple sites during August and September of 2017 within Springbrook National Park and Border Ranges National Park. Surveys generally occurred from late morning to early afternoon, with the dog deployed in the field no longer than two hours without a rest. The detection team surveyed freely, and the dog searched off‐leash.

Field trials in Springbrook National Park were located within the trapping grid employed for live and camera trapping aspects of this study and nearby areas (within 1 km of the trapping grid), which displayed presumed suitable habitat or climate requirements for *A. arktos* (Baker et al., 2014; Gray, Baker, et al., 2017). The detection dog surveyed all four transects, and the surrounding areas off‐leash.

The remaining locations used for canine field trials occurred throughout Border Ranges National Park. These locations represented areas where *A. arktos* is known to have historically occurred, including historical capture sites (Appendix 1) and where recent (unsuccessful) live trapping and camera surveys have been undertaken semi‐regularly (Baker et al., 2014; Gray, Baker, et al., 2017; Gray Burwell & Baker, 2016; Gray, Dennis, et al., 2017), as well as areas that displayed presumably suitable habitat and climatic requirements from which the species was unknown. As for Springbrook National Park, where possible, in Border Ranges National Park the detection dog team followed transects from a trapping grid employed in previous studies or surveyed freely in areas away from the trapping grid. The search focus was in and around several locations where voucher specimens had been historically retrieved (see Baker et al., 2014).

The detection team recorded the GPS location of each “indication” (i.e., each time the dog indicated that it had detected the odor and displayed a response), which included latitude, longitude, time, date, and elevation. During the field deployment, the detection dog was fitted with a GPS tracking collar to review search patterns after field deployments. The dog's indications were also marked by flagging tape for later confirmation of accuracy using camera traps. Any positive *A. arktos* indication by the dog was immediately investigated for the purposes of locating the scat or animal source of the odor.

### 2.6 Data analyses

Both camera trapping and live capture data were recorded in the form of repeated occurrence observations of a species, at fixed sites. A single site was defined as a local (1–2 m) region around a detection method, that is, either a fixed camera or Elliott trap. Therefore, multiple “sites” existed within the trapping grid. Sites were sampled for three nights over each of the four deployments. Data were pooled for each deployment at the trap level (i.e., three days of observations were pooled per deployment for each individual trap).

Occupancy modeling was conducted using the program PRESENCE version 2.12.24 (Hines 2006) for comparison of camera and live trapping methods for all species. Single‐season occupancy models (MacKenzie et al., 2002; Nichols et al., 2008) were employed in the current analysis to estimate the probability of detecting each species for each deployment with each method. Covariates used were as follows: deployment, species, and method.

Two models were run in PRESENCE to determine the effect of detection method, deployment variation, and species on detection probability. The first model constrained detection to be the same for both sampling methods, whereas for the second model detection was dependent on sampling method. The first model was,

$$\text{logit}\left(p\right)=\beta_{i}I\left(\text{deployment}==i\right)+\beta_{j}I\left(\text{species}==j\right),$$

and the second model was,

$$\text{logit}\left(p\right)=\beta_{i}I\left(\text{deployment}==i\right)+\beta_{j}I\left(\text{species}==j\right)+\beta_{k}I\left(\text{method}==k\right),$$

where,

$$\text{logit}\left(x\right)=\frac{e^{x}}{1+e^{x}},$$

is the logit function, *p* is the probability of detection, *β_i_* is the estimated parameter for the *i*th deployment, *β_j_* for the *j*th species and *β_k_* for the *k*th method, and,

$$I\left(x==i\right)=\left\{\begin{array}{c} x=i,\;1,\\ \text{else},\;0, \end{array}\right.$$

is the indicator function, which is used to encode the different categories from the data into the model. AICs were compared between models to determine the model that minimized the amount of information lost from the data.

## 3 RESULTS

The model that accounted for the variation in method was selected for analysis, as the AIC value (1,827.9) was lower than the AIC (2,163.04) for the model that did not (Table 1). This indicated that there was a better fit to the data for the model where detection was dependent on sampling method.

**Table 1:** Summary of model selection statistics for the two models using small mammal capture data from deployments

Model	AIC	deltaAIC	AIC wqt	No. of parameters
Constant detection	1,827.9	0.00	1.0000	10
Detection dependent on sampling method	2,163.04	335.14	0.0000	9

### 3.1 Live trapping general findings

The live trapping component consisted of a total of 2,400 trap‐nights, across all four deployments using Elliott traps (Table 2). A total of 784 captures were made during these deployments, representing five different species, including *Antechinus arktos*, *A. stuartii*, *Rattus fuscipes*, *Melomys cervinipes*, and *Perameles nasuta*. Because of the low number of captures of *P. nasuta* (1), it was not included in subsequent analyses.

**Table 2:** Summary of Elliott trapping data from Springbrook National Park for four deployments of Elliott traps between June and August 2017

Species	Total no. of captures	% of all mammal captures
*Antechinus arktos*	11	1.01
*Antechinus stuartii*	354	45.2
*Melomys cervinipes*	196	25
*Rattus fuscipes*	222	28.3
*Perameles nasuta*	1	0.001
Total captures	784	—

Note

Trap success % refers to the lowest and highest detection probability observed for each species across all four deployments. Capture refers to detecting the species in at least one photograph out of the three photographs taken in each “event.”

**Figure 3:**
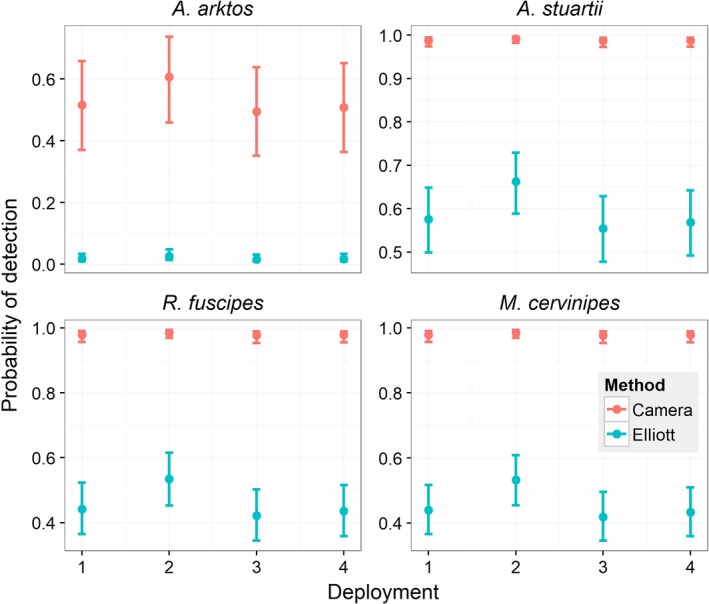
Relationship between detection probabilities estimated by single‐season occupancy models and detection method across all four deployments, for all four‐target species. Error bars represent 95% confidence intervals

Live trapping produced low detection probabilities for all four species (Figure 3). *Antechinus stuartii* was captured most often of all four species, contributing 45.2% of all mammal captures (Table 2), returning a detection probability between 55.5% and 66.3% across the four deployments. The second highest total number of captures was for *R. fuscipes* (28.3% of all mammal captures; detection probability between 42% and 53% across the four deployments), followed by *M. cervinipes* (25% of all mammal captures; detection probability between 42% and 53% across the four deployments) and lastly *A. arktos* (1.01% of all mammal captures; detection probability between 1.6% and 2.6% across the four deployments [Figure 3]). It should be noted that 8 of the 11 (73%) *A. arktos* captures during live trapping were from one male individual across multiple days. In all, four individual *A. arktos* were captured and PIT‐tagged: two females and two males. Only the one individual male was captured more than once.

### 3.2 Camera trapping general findings

**Figure 4:**
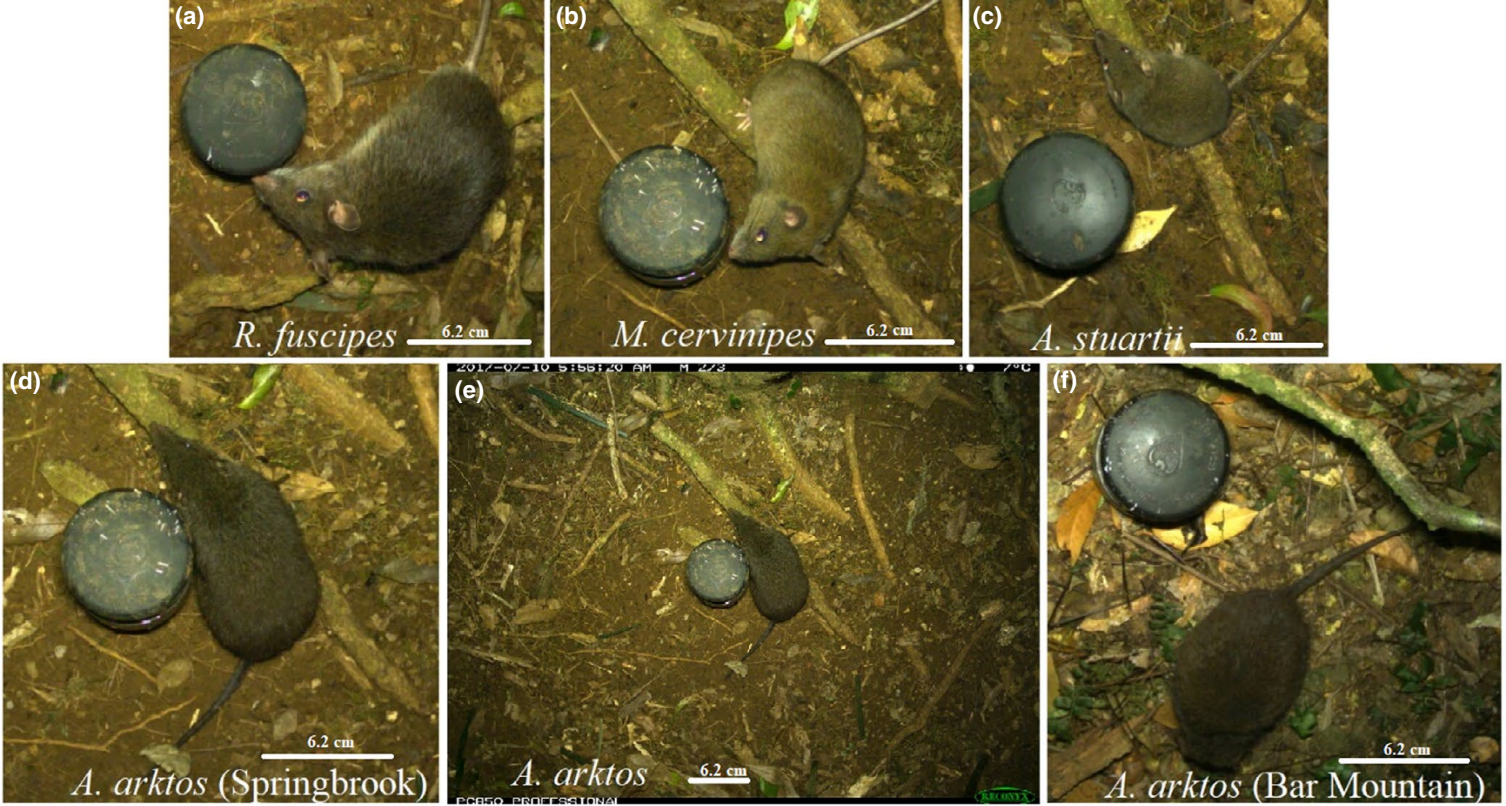
Cropped white flash camera photographs documenting all four small mammal species encountered during survey periods. Images were cropped for the purpose of the paper to ensure individual differences were observable. (a) Bush rat (*Rattus fuscipes*). (b) Fawn‐footed melomys (*Melomys cervinipes*). (c) Brown antechinus (*Antechinus stuartii*). (d) Black‐tailed dusky antechinus *(Antechinus arktos*) captured at Springbrook National Park. (e) Uncropped image of *A. arktos* pictured in (d), displaying detection grid size. (f) *A. arktos* captured at Bar Mountain, confirming canine‐based detections. Note the larger size, darker fur, and tail of *A. arktos* (d–f) compared to *A. stuartii* (c) and the rounded snout, larger ears, eyes, and pelage distinctions between the rodents (a, b) and antechinuses (c–f). Diameter of the blackened bait container is 6 cm

The camera trapping survey period consisted of 240 trap‐nights, across the total of four deployments (Table 3). Over all four deployments, mammal captures consisted of 3,893 photo events representing eight different mammal species, including four‐target species: *Antechinus arktos*, *A. stuartii*, *Rattus fuscipes*, and *Melomys cervinipes* (Figure 4). The remaining nontarget species were as follows: *Perameles nasuta*, *Trichosurus caninus*, *Thylogale thetis*, *Felis catus*, and multiple rainforest birds. Because of the inability to live trap these larger nontarget species, they were excluded from subsequent comparative analyses.

**Table 3:** Summary of white flash camera trapping data from Springbrook National Park for four deployments between June and August 2017

Species	Total no. of captures	% of all mammal captures
*Antechinus arktos*	74	1.9
*Antechinus stuartii*	1,173	30.1
*Melomys cervinipes*	1,121	28.8
*Rattus fuscipes*	1,066	27.4
*Perameles nasuta*	211	5.4
*Trichosurus caninus*	240	6.2
*Thylogale thetis*	5	0.12
*Felis catus*	3	0.07
Total mammal captures	3,893	—
Total Aves	7,763	—

Note

Trap success % refers to the lowest and highest detection probability observed for each species across all four deployments. Capture refers to detecting the species in at least one photograph out of the three photographs taken in each “event.”

Camera trapping for most species displayed high detection probabilities (Figure 3). *Antechinus stuartii* was captured most often of all four species, contributing 30.1% of all mammal captures (Table 3), returning a detection probability between 98.6% and 99.1% across the four deployments. The second highest total number of captures was for *M. cervinipes* (28.8% of all mammal captures; detection probability between 97.7% and 98.5% across the four deployments), followed by *R. fuscipes* (27.4% of all mammal captures; detection probability between 97.7% and 98.5% across the four deployments) and lastly *A. arktos* (1.9% of all mammal captures; detection probability between 49.4% and 60.7% across the four deployments [Figure 3]). Because of the high detection probabilities observed during the study, the camera trapping aspect appears to display an adequate sampling duration to detect a rare/ cryptic species such as *A. arktos*.

### 3.3 Detection dog findings

**Figure 5:**
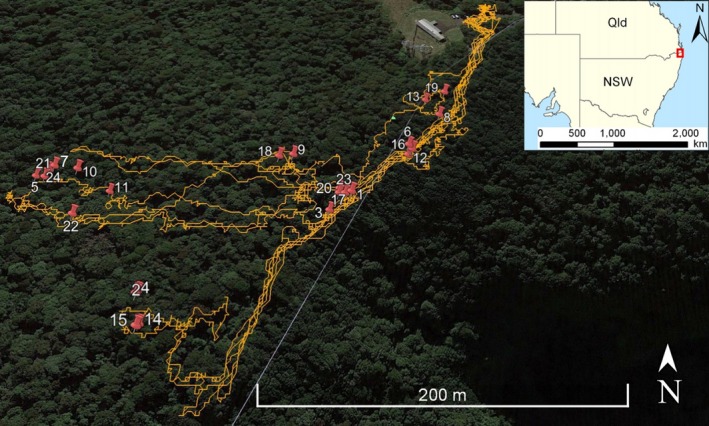
GPS tracking of detection dog in field surveys at Best of All Lookout walking track, Springbrook National Park, 2017, using a GPS collar. Red pins represent *A. arktos* odor indication, with corresponding numerical information for each indication corresponding to those in Table 4. Building in top of image represents repeater station, at Best of All Lookout car park area, located at the end of Repeater Station Road

**Figure 6:**
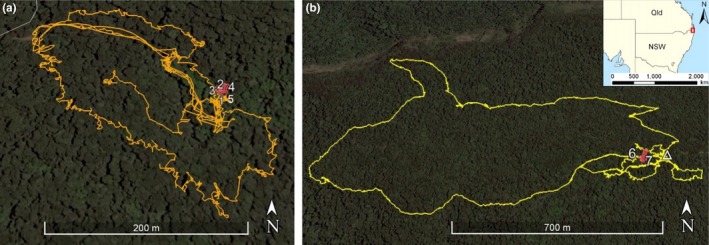
GPS tracking of detection dog in field surveys at Border Ranges National Park, 2017. (a) Left: Helmholtzia Loop Walking Track located on Brindle Creek Road. (b) Right: Bar Mountain Picnic Area, located on Bar Mountain Road, ∆ represents the Bar Mountain Picnic Area car park. Red pins represent *A. arktos* odor indication, corresponding information for each indication found in Table 5

Between August and September 2017, the detection dog team surveyed a total of 8 different sites across the three deployments. The detection dog indicated *A. arktos* odor a total of 31 times between the two national parks, 24 times within Springbrook National Park (Table 4, Figure 5), and seven times within Border Ranges National Park (Table 5, Figure 6). Because of the small size of the target species, and the inability to collect or identify any biological materials associated with the canine indications, white flash camera traps were used in combination to subsequently corroborate *A. arktos* presence at the indicated sites.

**Table 4:** Detection dog indications of *A. arktos* odor from field deployments in Springbrook National Park, 2017

Map no.	Indication site habitat
1	Tree trunk buttress root
2	Rock crevice
3	Hollow tree buttress root
4	Rock crevice
5	Buttress root cavity
6	*Helmholtzia glaberrima* base
7	Hollow log
8	Hollow log
9	Hollow tree buttress root
10	*Helmholtzia glaberrima* base
11	Boulders/*Helmholtzia glaberrima* base
12	Rock crevice
13	Rock surface
14	Rock crevice
15	Rock crevice
16	Rock crevice
17	Rock surface
18	Rock crevice
19	Buttress root cavity
20	Buttress root surface
21	Base of tree
22	Buttress root cavity
23	Rock crevice
24	Hollow log

Note

Map number corresponds to the pin locations shown in Figure 5. Indication site habitat corresponds to exactly where the detection dog indicated within the site.

**Table 5:** Detection dog indications of *A. arktos* odor from field deployments in Border Ranges National Park, 2017

Map no.	Indication site habitat
1	Among *Helmholtzia glaberrima*
2	Among *Helmholtzia glaberrima*
3	Along fallen log
4	Among *Helmholtzia glaberrima*
5	At buttress of tree
6	Around fallen timber next to dense vine thicket
7	Fallen log next to dense vine thicket

Note

Map number corresponds to the pin locations shown in Figure 6. Indication site habitat corresponds to exactly where the detection dog indicated within the site.

Within Springbrook National Park, three canine‐indicated sites away from the trapping grid were selected for camera corroboration. These three sites had indications which were in close proximity to each other (indication 14 and 15, 4 and 2, and 13 and 19, Table 4, Figure 5), and therefore, we could test six indication sites. Cameras were set as near as possible to the flagging tape (i.e., on the same tree as flagging tape, where possible) marking the dog's indication site and left for 28 days. Eight of nine cameras set in Springbrook National Park captured *A. arktos* at least once, with one camera documenting *A. arktos* 10 times in total.

Within Border Ranges National Park, the detection dog indicated seven times, with two of these indications adjacent to the Bar Mountain Picnic area (Figure 6a), and the remaining five indications at the Helmholtzia Loop Walking Track, at a previously established trapping grid for live trapping and camera trapping efforts (Figure 6b). The indications at the Helmholtzia Loop Walking Track and Bar Mountain Picnic area were similarly in close proximity, and thus, three cameras were set in the same area for 28 days, in an attempt to corroborate canine‐based indications. *Antechinus arktos* was captured on two of three cameras set surrounding the indication at the Bar Mountain Picnic Area (Figure 4). Camera traps from the Helmholtzia Loop Walking Track resulted in no captures of *A. arktos* during our camera deployment, but a subsequent study at the same dog detection site in December 2018 detected *A. arktos* on remote cameras.

## 4 DISCUSSION

The results demonstrate the utility of white flash digital camera traps for detecting and distinguishing coexisting, morphologically similar small mammal species, including species occurring at very low density, such as *A. arktos*. Features such as color/appearance, body size and shape, head size and shape, and tail length/appearance were more easily visualized in white flash (color) camera photographs when compared to infrared photographs, especially for *A. arktos* and *A. stuartii*. Both Elliott traps and camera traps permitted quick and confident species identification postcapture. However, when solely considering comparative probability of detecting the presence of all four common small mammal species in the study area, cameras proved superior to Elliott traps across a comparative 3‐day trapping effort. Similar studies comparing multiple detection methods also reported an advantage of remote camera traps compared to other detection methods. For example, Clare, McKinney, DePue, and Loftin (2017) found that remote cameras more readily detected *Martes americana* when compared to passive hair catches, and density estimates did not suffer from any negative bias because of an inability to distinguish the sex of an individual. Both Fisher, Heim, Code, and Paczkowski (2016) and Fisher and Bradbury (2014) similarly found that noninvasive genetic tagging via hair trapping constantly underestimated occupancy of *Ursus arctos* in one study, and *Martes americana, Pekania pennanti*, and *Gulo gulo* in the second, when compared to camera traps.

We could not control for potential multiple appearances of the same individual per night under cameras. However, our question of interest concerned the relative probability of detecting the presence of a species in a surveyed area. The fact that cameras remain an “open trap” throughout the night and are potentially less intimidating to target species than live capture traps are likely important influences on their efficacy in detecting species presence. Comparatively, Elliott traps can only survey one individual per processing effort. Researchers must consider this and other factors when selecting methods of detection.

The detection dog detected *A. arktos* at a range of locations within both present‐day and historical capture sites. Subsequent site‐specific white flash camera deployments corroborated the presence of *A. arktos* at canine‐indicated sites, including Border Ranges National Park, where presence of the species had not been confirmed for almost 30 years.

### 4.1 Mounting design and type of remote cameras

Camera‐based species detection relies implicitly on the ability to correctly identify a species and be confident of distinguishing it from similar taxa. Several studies have demonstrated the difficulties associated with identifying coexisting small mammal species from photographs taken using horizontally oriented camera traps (e.g., Glen et al., 2013; Meek & Vernes, 2016). One recent study found that horizontally positioned white flash digital camera traps produced photographs that did not provide enough precision of certain diagnostic features to enable accurate identification of some rodent species (Meek & Vernes, 2016). In contrast, for the current study, employing the revised vertical mounting design established by De Bondi et al. (2010) and adopted by Gray, Dennis, et al. (2017) permitted fast and confident identification of nearly all individual small mammal images to species level. In the current study, only three images could not be accurately identified to species level, whereas in Gray, Dennis, et al. (2017), a total of 334 infrared photographs were unable to be identified to species level (comprised of 134 mammals, 134 murid, and 66 *Antechinus* spp.).

Our vertical mounting design paired with a white flash camera produced dorsal photographs of individuals, which captured subtleties of many diagnostic features such as pelage color, guard hair length, body and head size, and tail length, shape, and color. If the typical horizontal mounting design for camera traps was employed in our study, most photographs would have instead captured the profile of an individual against dense background rainforest vegetation, and many resulting images would not have allowed for easy comparison of close‐ups. Similar issues were encountered by Meek et al. (2013), resulting in a lack of clarity when differentiating certain aspects of an individual from the background, which ultimately affected identification ability. However, a previous study employing a vertical mounting design found that even after clearing vegetation and leaf litter around the detection grid, difficulties remained in discriminating the tail of individuals from blades of grass or twigs (De Bondi et al., 2010). The current study avoided these problems by carefully clearing most grasses, leaf litter, and vegetation within the detection grid, leaving the ground relatively bare, which facilitated identification of individuals, especially in cases where only part of the animal's body was in frame. Such issues are habitat‐specific, and we were fortunate that rainforest leaf litter was easily manipulated, reducing handling time during camera deployment.

Gray, Dennis, et al. (2017) suggested that having prior knowledge of the target species within the study site provided a marked advantage when identifying individuals and for confirmation of identifications. We concur an element of live trapping is advantageous at any given site, allowing users to become acquainted with the appearance of a species in the hand, in real time. Representative individuals captured and identified by hand can also be released under deployed cameras to close the loop between appearances in the hand with those in a photograph. Such an approach further increases accuracy of identifications. As a final failsafe, the current study also utilized two different examiners to review all photographs and confirm identifications. We advocate this approach to improve accuracy of all camera‐based identifications.

Equally, to continue the improvement and development of camera trapping surveys, researchers must ensure that survey methods involve a clear emphasis on the fundamental processes of animal abundance, movement, and detection and should incorporate a more detailed treatment of methodological characteristics and assumptions. This clarity will enable future researchers to assess, compare, and develop the effectiveness and reliability of detection camera trapping surveys, further strengthening our ability to answer vital questions and close knowledge gaps (Burton et al., 2015).

### 4.2 Factors influencing trap success

Detection probabilities varied notably between detection methods. The probability of an individual being successfully detected ultimately depends on a multitude of factors. Effectiveness of a trap, the probability of an individual locating a trap, and the individual's reaction to the trap, among other factors, all have significant influences on trap success (Tasker & Dickman, 2001). In the current study, the observed variation in detection probabilities between species may have similarly been caused by additional factors such as bait strength, attraction to bait, trap‐shyness, or various biological factors influenced by population dynamics (Gray, Dennis, et al., 2017).

*Antechinus stuartii*, *M. cervinipes*, and *R. fuscipes* all displayed high detection probabilities within and across deployments for both trapping methods, relative to *A. arktos*. All three of the common species occur in high density within the trapping site and taken together represent the major components of terrestrial, small mammal taxa within subtropical rainforests of southeast Queensland (Wood, 1971). Thus, the relatively high probabilities of detection, regardless of trapping method, for these three small mammal species compared with that of *A. arktos* are unsurprising. However, there were some differences in relative detected species abundance between our study and previous work at the same site. In previous studies that employed live trapping or camera trapping methods within the same area, *R. fuscipes* produced the highest detection probabilities (Gray, Baker, et al., 2017; Gray, Dennis, et al., 2017). Live trapping components of the study performed by Gray, Baker, et al. (2017) occurred across a two‐year period, between April and October, and the camera trapping component of the study by Gray, Dennis, et al. (2017) was conducted across a three‐month period, between August and October. Yet in both camera trapping and live trapping regimes of the current study (late June–late August), *A. stuartii* displayed the highest detection probability and total number of captures, compared to *R. fuscipes* in the two previous studies. Breeding for Springbrook populations of *A. stuartii* occurs during the last 2 weeks of August, and it might be expected that activity and movement prior to this time would be increased, resulting in the relatively higher number of captures of this species observed here (Gray, Baker, et al., 2017; Leung, 1999; Wood, 1970). Gray, Baker, et al. (2017) found that *A. stuartii* exhibited no notable difference in trap success between years in their study, but they observed the highest trap success of this species in July. Field work for the current study occurred around the same period (all field work being completed during July, or within a few weeks before or after July).

*Antechinus stuartii* also displayed a decrease in % total mammal captures between the two detection methods in the current study. *Antechinus stuartii* was the smallest species to appear in both detection methods and may have sometimes been deterred from investigating bait containers, or chased away by larger, more dominant species. In previous studies, *A. stuartii* has exhibited a heightened alertness when more dominant species are present and even fled when certain species approached (Dickman, 1991; Gray, Dennis, et al., 2017). In the present study, similar behaviors may have occurred between *A. stuartii* and the larger species, *R. fuscipes*, *A. arktos* and *M. cervinipes*, at least in part accounting for reduced relative incidence of *A. stuartii* in camera traps versus Elliott traps.

For detection of more common species (*A. stuartii*, *R. fuscipes*, and *M. cervinipes*), the survey period of three days was sufficient. For infrared camera traps, Gray, Dennis, et al. (2017) suggested one deployment (consisting of 11 days) to achieve adequate detection probabilities of the three common mammal species, deploying 11 cameras. However, longer deployments naturally increase time, cost, and effort in terms of image processing and bait refreshment (but will not alter the time it takes to set up and pack up camera traps). Gray, Dennis, et al. (2017) also suggested replacing bait every three days to maximize efficiency, and survey periods of 11 days would therefore include having to rebait traps multiple times throughout the deployment. For future studies aimed at locating these three species with white flash camera traps, we advocate a single deployment of a greater number of cameras, across the same size grid, following the same three‐day duration. In designing and resourcing any camera study, a reduced deployment duration must naturally be traded off against higher number and cost of camera units. However, because of the vast array of information that cameras provide, they may allow for reductions in costs elsewhere. For example, Clare et al. (2017) demonstrated that cameras were able to provide a vast reduction (more than 50%) in laboratory analysis of hair samples from hair catches, since using cameras increased their ability to determine nontarget hair samples, consequently allowing researchers to recoup their initial camera investment. Similarly, in cost efficiency alone, Clare et al. (2017) established remote cameras to be the most cost‐efficient method for density estimation of marten (*M. americana*) populations.

During the Elliott trapping regime of the present study, we noted traps that had obviously been disturbed (moved, kicked, bag removed, coconut husk pulled from trap, etc.). Both *P. nasuta* and *A. lathami* were regularly observed disturbing the traps over the course of the study period (M. L. Thomas, personal observation). Along with interference by *T. caninus*, these species are expected to be the main cause of disturbances. Disturbance for days one and two of “deployment A” was not recorded. However, in total across all four deployments, 503 traps were disturbed. This constitutes 21% of all traps across the four deployments being disturbed, which has a notable impact on the resulting data for that study area by substantially reducing trapping potential of target species. This aspect, paired with the potential for Elliott traps to capture only one individual per night, are contributing factors explaining the difference in detection probabilities between methods.

### 4.3 Detection dog efficacy in surveying small, cryptic mammal species

Detection dogs have been employed in past studies for locating rare or elusive species with high success (Duggan, Heske, Schooley, Hurt, & Whitelaw, 2011; Reindl‐Thompson et al., 2006). Our study clearly corroborates the utility of conservation detection dogs in locating cryptic and rare species, such as *A. arktos*, where other methods such as live and camera trapping have previously failed. Successful identification of *A. arktos* presence by the detection dog was expected within the Best of All Lookout site at Springbrook National Park. *Antechinus arktos* was known to occur there, having been captured in both Elliott and camera traps in trapping regimes prior to dog deployment in the present study and previously. However, the detection dog coupled with subsequent site‐specific camera deployment enabled us to explore and corroborate presence of *A. arktos* outside the trapping grid at this location. *Antechinus arktos* is now known to occur within habitat spanning the entirety of the walking track at the Best of All Lookout. Sixty‐six percent of all canine‐based indications at the Springbrook National Park site were outside of the trapping grid, with the majority of these indications located around the walking track. The dog also exhibited a remarkable ability from the first field deployment to discern between *A. arktos* and *A. stuartii*, when the latter were more common in study sites by orders of magnitude.

The present research presents a successful implementation of a two‐stage survey strategy, using vertically oriented white flash cameras as a follow‐up to canine detection. The addition of cameras placed strategically around microhabitat sites indicated by the detection dog enabled successful corroboration (on eight of nine cameras) of *A. arktos* presence and activity at specific locations. Employing this survey strategy increases time and effort, requiring extra field time (deployment, processing, rebaiting, etc.) and data management, but has positive implications for success of conservation detection dogs in locating cryptic or rare species, especially when biological material cannot be easily collected.

*Antechinus arktos* presence was confirmed by the detection dog in Border Ranges National Park after no confirmed sightings or captures in the region for the last 30 years. Taken together, over 5,000 trap‐nights have been undertaken at Border Ranges National Park since the last sighting of *A. arktos* in 1988 and have resulted in zero captures or sightings of *A. arktos* (Baker et al., 2014; Gray et al., 2016). The current study employed both Elliott and camera trapping in these regions of the Border Ranges at a previously established trapping grid along the Brindle Creek site (3 nights of 20 cameras, 600 Elliott trap‐nights) and camera trapping at the Bar Mountain Picnic Area (3 nights of 20 cameras). No *A. arktos* were recorded. However, following dog deployment, cameras placed 1–3 meters from pinpointed canine detections at Bar Mountain, within 30 m of existing camera trapping grids, successfully detected *A. arktos* on the first night of deployment.

This suggests some small mammal species persist at very low density and may remain undetected even after intensive and strategic surveys using concerted live and camera trapping. Presumably, at Bar Mountain Picnic Area, our dog detected an *A. arktos* either at its subterranean nest, or based on scent overlaying, a recently used foraging trail. Such site‐specific microhabitat use proved the difference in targeting the exact camera location to capture footage of an *A. arktos* the following night.

### 4.4 Management implications

The development and growing understanding of detection methods has provided a variety of novel opportunities for gathering a plethora of ecological and biological information. Information provided by these detection methods may consequently prompt changes to management or policy. Therefore, without strategic selection of method and experimental design, researchers may encourage the use of survey methods or monitoring designs that fail to effectively complete the objective or detect the target species (Clare et al., 2017). The choice of detection method will primarily depend upon the management objectives, as each method exhibits its own specific benefits and drawbacks, so the detection method used should be survey specific. Our findings demonstrate the utility of white flash, vertically oriented, remote wildlife camera traps and conservation detection dogs when compared to the traditional method of live trapping (Elliott traps) for locating rare and cryptic species. Detection dogs provide a unique opportunity to locate target species, which may be overlooked by traditional methods. The ability of dogs to provide more detailed, fine‐scale information (e.g., between sexes or even individuals) warrants further exploration. If we are to mitigate the risks that endanger our mammals, it is imperative that we successfully survey rare species to enable the development of effective conservation, management, and monitoring programs. Indirect methods, such as detection dogs, will become an increasingly important tool in achieving this goal.

## CONFLICT OF INTEREST

None declared.

## AUTHOR CONTRIBUTIONS

Morgan L. Thomas, Andrew M. Baker, and Lynn Baker conceived the study. Morgan L. Thomas and Lynn Baker acquired the data. Morgan L. Thomas and James R. Beattie involved in formal analysis and interpretation of the data. Morgan L. Thomas, Andrew M. Baker, Lynn Baker, and James R. Beattie designed the methodology. Morgan L. Thomas involved in project administration. Andrew M. Baker supervised the study. Morgan L. Thomas, Lynn Baker, James, R. Beattie, and Andrew M. Baker involved in validation process. Morgan L. Thomas visualized the study. Morgan L. Thomas wrote the original draft. Morgan L. Thomas, Lynn Baker, James, R. Beattie, and Andrew M. Baker wrote, reviewed, edited, and approved the final draft of the manuscript.

## Data Availability

All observational (Camera trap/Elliott trap) data for this study will be available from the Dryad Digital Repository, at: https://doi.org/10.5061/dryad.7h44j0zqr.

## Appendix 1

**Table A1:** Historical captures of *A. arktos* within Border Ranges National Park which were surveyed by the detection dog in the current study, including latitudes and longitudes, no indication by dog of *A. arktos* presence

Year/map no.	Latitude	Longitude	Indication
1980—8	28°22′32.02″S	153°6′22.55″E	None
1980—9	28°22′36.95″S	153°4′6.04″E	None
1995—10	28°25′0.22″S	153°7′8.15″E	None
